# Medical Organization

**Published:** 1873-05

**Authors:** 


### EDITORIAL AND MISCELLANEOUS.

### MEDICAL ORGANIZATION.

The harmonious Annual Association of medical men in State and National bodies, for purposes of professional counsel and mutual improvement, is as yet an unsolved problem in the Western world. Whether or not it be an unsolvable problem, as well, remains for the future to disclose.

It is asserted by some that medical men are not, by nature, *gregarious* animals, and herein is supposed to rest the root of the difficulty. Others maintain that science and letters tend always to aristocracy; that they are, in essence, opposed to agrarian democracy, and cannot flourish under the rule of a strictly professional republicanism.

That physicians, as a class, are sadly afflicted with professional *hyperæsthesia*, is undeniable;—that they are, in common with mankind in general, more or less swayed by innate *selfishness*, is a trite truism. That the dissensions and discords in our collective bodies are due, in part at least, to such weaknesses, may be fairly asserted.

The number of individuals whose fertile minds have improvised panaceas for these acknowledged ills, is by no means small; and, upon the other hand, the few who have given the subject deep and anxious thought, seem well nigh despairing of remedy.

We would not presume so much as to suggest our panacea, even though we had one—nor do we propose to attempt either diagnosis or prognosis of the formidable malady, but simply to chronicle some of the efforts of others who have the matter at heart.

In a late number of the *Philadelphia Medical Times*, Prof. S. D. Gross gives expression to his views as to the remedy for the defects in the working of the American Medical Association. We extract the following suggestions:

"The alterations in our constitution, calculated, in my opinion, to meet these contingencies, are: First—The appointment of a medical council, consisting of not less than ten members, a majority of whom shall be residents of the place of meeting, to whom shall be referred all applications for membership, all questions of ethics, and all other business matters, and whose decision shall be final. Such a body, composed solely of wise, judicious and experienced men, sitting as a kind of court, or board of censors, should hold office for only one year, elect its own President and Secretary, and hold quarterly meetings, the last not earlier than two days before the annual meeting of the Association. Secondly—The Association should consist exclusively of members of State societies, who shall be elected at the annual meetings of such societies; the number of delegates to be optional, but not less, if possible, than twenty-five or thirty. They should hold office for one year, but their attendance should entitle them to permanent membership, and to a vote upon every question in the Association.

"If the *outside* affairs of the Association were managed by a properly organized council, such as is here contemplated, the result would be to prevent the introduction of a vast amount of irrelevant and objectionable matter, which, in the existing state of things, is a source of so much annoyance and irritation, and which so greatly interferes with the deliberations of an assembly of a purely professional character. No society of this kind can long survive if it permits the introduction and discussion of political questions. Medical politicians are not fit representatives of the interests of the medical profession. We want no such men among us."

At the March meeting of the Medical Association of the State of Alabama, a new constitution was adopted, in which certain steps were taken in the direction we have indicated above.

*First*—The membership is subdivided into four classes, namely: "Members," "Delegates," "Counsellors," "Correspondents."

It was deemed best to recognize at once an element of aristocracy by the establishment of a house of counsellors, limited to one hundred, who are alone entitled to hold office in the Association. Counsellors and delegates alone can vote. Ingress to the house of counsellors is by two-thirds vote of the house, and egress is by death, resignation, or impeachment. Counsellors, both present and absent, are taxed ten dollars annually. A failure to pay this assessment, or absence from three consecutive meetings of the Association, forfeits the Counsellorship.

*Secondly*—A board of five censors, like other officers chosen from the counsellors, with an official tenure of five years, one to be replaced annually. The functions of the board of censors are those of a general committee of reference in all questions relative to the organization and general welfare of the Association, and a court of impeachment, to try all charges and appeals. They report their action to the Association for approval.

*Thirdly*—All elections are by ballot and *without nomination*, as in masonic lodges, balloting being continued until election is had.

At the April meeting of the Medical Association of the State of Georgia, a new Constitution was likewise adopted, which also provides a board of censors, consisting of five members, with a tenure of five years, one to be replaced annually. The censors decide who are entitled to seats in the Association, report upon all applications of membership, and act as a court of impeachment for trial of charges. They report their action to the Association for ratification or rejection. B.

---

